# RNase HI Is Essential for Survival of *Mycobacterium smegmatis*

**DOI:** 10.1371/journal.pone.0126260

**Published:** 2015-05-12

**Authors:** Alina E. Minias, Anna M. Brzostek, Malgorzata Korycka- Machala, Bozena Dziadek, Piotr Minias, Malini Rajagopalan, Murty Madiraju, Jaroslaw Dziadek

**Affiliations:** 1 Institute of Medical Biology, Polish Academy of Sciences, Lodz, Poland; 2 Department of Immunoparasitology, University of Lodz, Lodz, Poland; 3 Department of Teacher Training and Biodiversity Studies, University of Lodz, Lodz, Poland; 4 Department of Microbiology, University of Texas Health Center at Tyler, Tyler, Texas, United States of America; University of Delhi, INDIA

## Abstract

RNases H are involved in the removal of RNA from RNA/DNA hybrids. Type I RNases H are thought to recognize and cleave the RNA/DNA duplex when at least four ribonucleotides are present. Here we investigated the importance of RNase H type I encoding genes for model organism *Mycobacterium smegmatis*. By performing gene replacement through homologous recombination, we demonstrate that each of the two presumable RNase H type I encoding genes, *rnhA* and MSMEG4305, can be removed from *M*. *smegmatis* genome without affecting the growth rate of the mutant. Further, we demonstrate that deletion of both RNases H type I encoding genes in *M*. *smegmatis* leads to synthetic lethality. Finally, we question the possibility of existence of RNase HI related alternative mode of initiation of DNA replication in *M*. *smegmatis*, the process initially discovered in *Escherichia coli*. We suspect that synthetic lethality of double mutant lacking RNases H type I is caused by formation of R-loops leading to collapse of replication forks. We report *Mycobacterium smegmatis* as the first bacterial species, where function of RNase H type I has been found essential.

## Introduction

RNases H are ubiquitous enzymes present is eukaryotes, prokaryotes, archaeons and viruses. They are involved in the removal of RNA from RNA/DNA hybrids during DNA replication, repair and transcription. Based on the differences in their amino acid sequences, RNases H have been divided into two types and three classes. Prokaryotic RNase HI or eukaryotic H1 represent RNases H type 1, while prokaryotic RNases HII and HIII or eukaryotic RNases H2 represent RNases H type 2.

**Fig 1:**
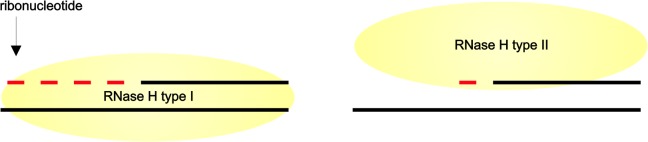
Schematic representation of a mode of action of RNases H. RNases H cleave RNA from the RNA/DNA duplex. RNases H type I recognize two strands of the heteroduplex and cleave RNA when at least four ribonucleotides are present. In contrast, RNases H type II cleave even single ribonucleotides and recognize transition from DNA to RNA on a single strand.

Type 1 RNases H require at least four ribonucleotides as a substrate for a cleavage to occur [1,2] and they do not prefer any consensus sequence to perform this reaction [1]. *In vitro* studies showed that introducing various modifications that decrease DNA flexibility in RNA/DNA duplex abrogates cleavage by RNase H1 [3]. Thus, the enzyme must recognize both RNA and DNA strands. This observation was confirmed by obtaining co-crystal structure of RNase HI of *Bacillus halodurans* in complex with RNA/DNA [4]. In contrast, RNases H type II recognize and cleave single ribonucleotides within RNA/DNA duplex and are thought to recognize the transition from deoxiribonucleotides to ribonucleotides on a single strand [5,6] (Fig 1).

RNases H type I recognize two substrate types. The first type is RNA primers generated during DNA synthesis. The second type is R-loops: a three strand nucleic acid structures consisting of an RNA/DNA hybrid and a displaced DNA strand. It is thought that such loops occur as a consequence of transcription, when a nascent transcript anneals to the matrix DNA. The presence of R-loops has been reported in several processes, including DNA replication [7–9], transcription termination [10], regulation of gene expression [11,12] and other processes specific to eukaryotes [13–15]. R-loops can achieve considerable length, as they for example exceed 1 kbp at immunoglobulin class switch region [15]. In fact, RNases H type I are thought to be likely to act on relatively stable, transcription associated R-loops [16,17].

To date, higher eukaryotes have been shown to possess one RNase H type 1 and one RNase H type 2 (composed of three subunits). Both have been shown essential for survival, even at the stage of embryogenesis [18,19]. *Saccharomyces cerevisiae* also possesses two RNases H–one RNase H type 1 and one RNase H type 2 composed of three subunits–but both are dispensable for growth [20]. In contrast, the number of RNases H encoded in bacterial genomes is more variable [21], and more complicated in terms of being essential for viability. For example, *Escherichia coli* possesses one RNase HI and one RNase HII [22], while the genome of *Bacillus subtilis* contains two RNase H type 2- RNase HII and RNase HIII [23]. In both of these species, there are contradictory reports regarding essentiality of RNase H enzymes [24–31], however, most recent studies show that they are dispensable under certain conditions [25–27,30,31]. RNase HI has also been shown dispensable in *Haemophilus influenzae* [32]. The genome of *Mycobacterium tuberculosis* contains one gene encoding an RNase H type II, *rnhB* [33], and one gene encoding a bifunctional protein, Rv2228c. Its N-terminal domain is homologous with eukaryotic and prokaryotic RNases H type I, while C-terminal domain is homologous with alpha ribazole phosphatase (CobC) involved in cobalamin (vitamin B12, B12) biosynthesis [34]. Recombinant protein expression confirmed the activity of both domains *in vitro* (in an artificially set up reaction between the enzyme and the substrate) [34]. In turn, the genome of *Mycobacterium smegmatis* seems to encode four RNases H. Two of them belong to RNases H type I. The first one is encoded by *rnhA* and the RNase HI activity of the derived protein has been confirmed *in vitro* [35]. The second is a homolog of Rv2228c of *M*. *tuberculosis*, MSMEG4305. Further, the genome of *M*. *smegmatis* encodes an RNase H type II, through gene *rnhB*. Additionally, the gene MSMEG5849 of *M*. *smegmatis* encodes a protein which presents RNase HII activity through domain Duf429 (until recently referred to as a domain of unknown function) [36]. A protein with limited homology to Duf429 can be found in *M*. *tuberculosis*. The summary of presumable RNase H encoding genes in *E*. *coli*, *M*. *tuberculosis* and *M*. *smegmatis* is presented in Table 1.

**Table 1:** Summary of presumable RNases H of *E*. *coli* K12_MG1655, *M*. *tuberculosis* H37Rv and *M*. *smegmatis* mc^2^ 155.

Type of RNase H	*E*. *coli* K12_MG1655	*M*. *tuberculosis* H37Rv	*M*. *smegmatis* mc^2^ 155
I	RnhA	Homolog is absent	RnhA
I	Homolog with RNase HI domain is absent; CobC (homology with acid phosphatase domain)	Rv2228c	MSMEG4305
II	RnhB	RnhB	RnhB
II	Homolog is absent	MT0800 (homology with Duf429 domain)	MSMEG5849

The aim of this study was to understand the importance of RNases H type I for *M*. *smegmatis*. *M*. *smegmatis* is a model organism to study *Mycobacterium* genus, which includes *M*. *tuberculosis*. We demonstrate that deletion of both RNases H type I encoding genes leads to synthetic lethality. Further, we demonstrate that deletion of each RNase H type I did not alter growth rates of the mutants. Finally, we question the possibility of existence of RNase HI related alternative mode of initiation of DNA replication in *M*. *smegmatis*, the process initially discovered in *E*. *coli*. We suspect that RNases H type I may be essential for the survival of *M*. *smegmatis* due to formation of R-loops, which when unresolved, collapse replication forks.

## Materials and Methods

### *In silico* analysis

Genes presumably encoding RNases H in *M*. *smegmatis* mc^2^ 155, *E*. *coli* K12_MG1655 and *M*. *tuberculosis* H37Rv were identified and obtained from National Center for Biotechnology Information (NCBI) database. The span of the domains was defined using Simple Modular Architecture Research Tool (SMART) [37]. The homology between the domains was estimated using Basic Local Alignment Search Tool (BLAST) of NCBI. The alignments were visualized using MultiAlin [38] and ESPript 3.0 [39].

### Bacteria and culture conditions

Cultures of *E*. *coli* T10 were carried out at 37°C for 18–20 h in liquid Luria-Bertani broth, and when necessary supplemented with antibiotics or other supplements or both at the following concentrations: kanamycin (Bioshop) 50 μg/ml; ampicillin (Bioshop) 100 μg/ml; X-gal (Sigma) 40 μg/ml, IPTG 0.4 mM (Sigma). Cultures of *M*. *smegmatis* during gene replacement were carried out in nutrient broth (NB) (Difco) or 7H9 broth (Becton, Dickinson and Company) with or without oleic albumin dextrose catalase growth supplement (OADC) (Becton- Dickinson) and 0.05% Tween 80 (Sigma) at 37°C or 28°C. When necessary, media were supplemented with antibiotics or other supplements or both at the following concentrations: kanamycin (Sigma) 25 μg/ml; X-gal (Sigma) 40 μg/ml, 0.4% succinate (Sigma), sucrose 2% (Sigma), vitamin B12 10 μg/ml (Sigma). A list of *M*. *smegmatis* strains used in this study is presented in Table 2.

**Table 2:** List of *M*. *smegmatis* strains used in this study.

Strain	Characteristics
*M*. *smegmatis* mc^2^ 155	Reference strain
∆*rnhA*	*rnhA* deletion mutant of *M*. *smegmatis* mc^2^ 155
∆4305	MSMEG4305 deletion mutant of *M*. *smegmatis* mc^2^ 155
∆*rnhA*/∆4305*attB*::*Pami-rnhA*	derivative of *M*. *smegmatis* mc^2^ 155 carrying deletions within *rnhA* and MSMEG4305 complemented with full *rnhA* gene under acetamide promoter at *attB* site, Hyg^R^
∆*rnhA*/∆4305*attB*::*Pami*-4305	derivative of *M*. *smegmatis* mc^2^ 155 carrying deletions within *rnhA* and MSMEG4305 complemented with full MSMEG4305 gene under acetamide promoter at *attB* site, Hyg^R^
∆*rnhA*/∆*dnaAattB*::*dnaA*	derivative of *M*. *smegmatis* mc^2^ 155 carrying deletions within *rnhA* and *dnaA* complemented with full *dnaA* gene under natural promoter at *attB* site, Gm^R^, Hyg^R^

For determination of growth rates bacterial cells were transferred to fresh NB medium and cultured until the cultures reached OD_600_ between 0.6 and 0.9. Aliquots of these seed cultures were inoculated in fresh 7H9 broth supplemented with OADC at starting OD_600_ = 0.05. The cultures were incubated at 37°C with vigorous shaking for 48 h. At desired intervals of time samples of cultures were harvested and analyzed using spectrophotometer (Pharmacia Biotech Ultrospec 2000). To assess the number of the colony forming units, samples were serially diluted in fresh NB broth and plated on non-selective NB medium, which were incubated at 37°C until obtaining visible colonies. Each experiment was performed at least in triplicate. For determination of cell length drops, the cultures harvested from 24h cultures were placed on glass slides, fixed in flames and analyzed on Nikon Eclipse TE2000 microscope.

### M. smegmatis mutants

The mutants were obtained by using gene replacement protocol as previously described [40–42]. The procedure required construction of gene replacement plasmids and complementation plasmids. Primers used for obtaining the mutants are listed in Table 3.

**Table 3:** Primers used in this study.

Construction of plasmids used for gene replacement					
Name	Sequence	Primer pair	Template	intro-duced res-triction sites	Purpose
MsGR1rnhA	TCGAGGGCAAGCTGCGCGAC	MsGR2rnhA	mc^2^ 155	-	gene replacement *rnhA*
MsGR2rnhA	CGTAGCACCGCACCCCAGCC	MsGR1rnhA	mc^2^ 155	-	gene replacement *rnhA*
MsGR3rnhA	CGGGATCCGTGCGCGCGCCACCAGGTC	MsGR4rnhA	mc^2^ 155	BamHI	gene replacement *rnhA*
MsGR4rnhA	GAAGCTTCCGCGAGGGGCCGAACACC	MsGR3rnhA	mc^2^ 155	HindIII	gene replacement *rnhA*
GR1MsRnhAII-KpnI	GGTACCCCGCCGACGATGATGCTGTC	GR2MsRnhAII-BamH	mc^2^ 155	KpnI	gene replacement MSMEG4305
GR2MsRnhAII-BamH	CAAGCGGCGCAACGGGATC	GR1MsRnhAII-KpnI	mc^2^ 155	-	gene replacement MSMEG4305
GR3MsRnhAII-BamH	CGGGATCCTACACAACCGCGCCGTAGCC	GR4MsRnhAII-Hind	mc^2^ 155	BamHI	gene replacement MSMEG4305
GR4MsRnhAII-Hind	GCAAGCTTTCGCTGCTGGGTGCCGTGAC	GR3MsRnhAII-BamH	mc^2^ 155	HindIII	gene replacement MSMEG4305
GmBstBs	CTTCGAAGGCTGACGGAATTTATGCCTCTTC	GmBstBr	pINT3	BstBI	gentamycin resistance cassette
GmBstBr	CTTCGAACAGGAATCGAATGCAACCGG	GmBstBs	pINT3	BstBI	gentamycin resistance cassette
MsA1-5562-BglIIs	CAGATCTGTGAACCACCGGCACCACGCC	MsA1-5562-XbaI	mc^2^ 155	BglII	complementation *rnhA*
MsA1-5562-XbaI	CTCTAGATGGTGGTCGGCCTGGCGGG	MsA1-5562-BglIIs	mc^2^ 155	XbaI	complementation *rnhA*
MsrnhAIIPace-sBglII	CAGATCTGTGAAGGTTCTCGTCGAGGCCGAC	MsrnhAII-rev-Xba	mc^2^ 155	BglII	complementation MSMEG4305
MsrnhAII-rev-Xba	CTCTAGATGCACTCGTGAGCTACAGGTACGC	MsrnhAIIPace-sBglII	mc^2^ 155	XbaI	complementation MSMEG4305
MsdnaA+PrHindIIInat	CTGTCGATCAGACGCGCCCAC	MsrnhAII-rev-Xba	mc^2^ 155	-	complementation *dnaA*
MsdnaA+PrXbaIrev	CTCTAGATCTCCGAGCTCAGCGTTTGGC	MsdnaA+PrHindIIInat	mc^2^ 155	XbaI	complementation *dnaA*
Construction of plasmid for recombinant protein expression					
Name	Sequence	Primer pair	Template	intro-duced res-triction sites	purpose
RnhA-f	CGAATTCGTGAACCACCGGCACCACGCC	RnhA-r	mc^2^ 155	EcoRI	recombinant protein
RnhA-r	CAAGCTTGGTGGTCGGCCTGGCGGG	RnhA-f	mc^2^ 155	HindIII	recombinant protein
Southern blotting					
gene name	primer name	Sequence	primer pair		restriction enzyme
*rnhA* before comple-menta-tion	coDCOH1s	GTGAACCACCGGCACCACGCC	coDCOH1r		PvuI
*rnhA* before comple-menta-tion	coDCOH1r	GGCGGGCAACAAGCTCAACGG	coDCOH1s		PvuI
*rnhA* after comple-menta-tion	MsGR3rnhA	CGGGATCGGTGCGCGCGCCACCAGGTC	MsRnhA1probe-r-delikom		BamHI, PvuI, XbaI
*rnhA* after comple-menta-tion	MsRnhA1probe-r-delikom	TGTGGGCCGGTGCGGTGG	MsGR3rnhA		BamHI, PvuI, XbaI
MSMEG4305	Ms4305-probe-s	GACAACGACGCCAGGTCCAGG	Ms4305-probe-r		PvuI
MSMEG4305	Ms4305-probe-r	GTGAAGGTTCTCGGTCGAGGCCG	Ms4305-probe-s		PvuI
*dnaA*	Msdnaprobe-s	GCAAGAAGGCGCAGATGGATCG	MsdnaAprobe-r		ClaI, HindIII
*dnaA*	MsdnaAprobe-r	GCGGATCTTCTTCTCGGCGTACATC	Msdnaprobe-s		ClaI, HindIII

Briefly, for gene replacement purpose, the sequences flanking desired deletion were amplified by PCR and consecutively introduced into p2NIL plasmid, followed by introduction of PacI suicidal cassette excised from pGOAL17 vector. For complementation under an acetamide promoter, a native copy of the gene of interest was amplified by PCR and introduced into pJAM2. The gene was next excised from the pJAM2 together with an acetamide promoter and introduced into pMV306Hyg. All cloning was performed in *E*. *coli* Top10 cells (Invitrogen). A plasmid allowing deletion in *dnaA*, previously used in [43], was modified by addition of gentamycin resistance cassette within BstBI restriction site. Plasmids were introduced into mycobacterial cells, which were further subjected to multistep selection process allowing detection of the mutants. Presence of intact copy of the *dnaA* gene within the ∆*rnhA*/∆*dnaAattB*::*dnaA* strain at the *attB* site was confirmed by sequencing.

### Exchange of complementation vectors with genetic cassettes for an empty vector

Conditional mutants carrying a copy of a presumably essential gene were electroporated with an empty pMV306Km plasmid. For exchange of complementation vectors with genetic cassettes for an empty vector (ExEV) on rich medium, bacteria after transformation were inoculated in NB broth and cultivated overnight at 37°C with vigorous shaking. Afterwards, bacteria were plated on NB selective medium supplemented with Km and cultivated up to one week at 37°C. For ExEV on minimal medium, bacteria after transformation were inoculated in 7H9 broth without the addition of OADC and cultivated overnight at 28°C with vigorous shaking. Afterwards, bacteria were plated on 7H10 medium without the addition of OADC supplemented with Km and cultivated for up to three weeks at 28°C. In case of ExEV on cassettes containing MSMEG4305, an additional variant of the experiment was performed which included supplementation of both rich and poor media with B12. Clones growing on Km containing media were screened in search of an intact version of the gene of interest. Encountering a clone devoid of an intact version of the gene would mean that the gene is non-essential. On the other hand, inability to find such clones would confirm the essentiality of the gene.

### Southern blotting

Primers used for production of hybridization probes and restriction enzymes used for digestion of genomic DNA are listed in Table 3.

### Recombinant RnhA expression and purification

Sequence encoding RnhA, identified in NCBI database, was amplified by PCR (primers listed in Table 3) and introduced into pHIS2 expression plasmid. Cloning was performed in *E*. *coli* Top10 cells (Invitrogen). The plasmid was further introduced into *E*. *coli* BL21. Expression of the protein was performed at 37°C overnight in the presence of 0.4 mM IPTG. The pellet was resuspended in 10 ml of Binding Buffer (50 mM Tris-HCl pH 8 (Sigma); 6–8M urea (Sigma)) and sonicated in short bursts (Bioblock Scientific Vibracell). The addition of urea was necessary to denature and solubilize otherwise insoluble protein. Next, the sample was incubated for 2 h with mild shaking. The sample was centrifuged at 17000 x g at 12°C for 30 min. The supernatant was transferred through filtered syringe (0.45 μm, Millex) and placed on affinity column containing Ni-NTA resin (ThermoScientific). Following binding of the protein to the Ni-NTA resin, the column was washed with Binding Buffer and Wash Buffer (60 mM imidazole (Sigma), 0,4 M NaCl (Sigma), 20 mM Tris- HCl pH 8 (Sigma)). Next, the recombinant protein was washed out with Elution Buffer (1M imidazole (Sigma), 0.5 M NaCl (Sigma), 20 mM Tris- HCl pH 8 (Sigma)). The sample containing recombinant protein was concentrated on a Novagen concentrator until achieving the final concentration of 1 mg/ml. The protein was used to immunize rabbits and for production of polyclonal antibodies.

### Production of anti- RnhA antibodies

Laboratory New Zealand rabbits were raised under standard conventional conditions in the approved by Polish Ministry of Science and Higher Education animal facility of the Institute Microbiology, Biotechnology and Immunology, Faculty of Biology and Environmental Protection, University of Lodz and were used for the immunization experiments. The experimental procedures were approved and conducted according to guidelines of the appropriate Polish Local Ethics Commission for Experiments on Animals No. 9 in Lodz (Agreement 54/ŁD1/2011).

### Western blotting

Bacteria were cultured in NB medium until OD_600_ reached between 0.6 and 0.9. Aliquots of these seed cultures were inoculated in fresh 7H9 broth starting OD_600_ = 0.05. The cultures were incubated at 37°C with vigorous shaking overnight. The following morning 5 ml of each culture were spun down at 4°C at 8000 x g and the pellet was resuspended in 1 ml TE (10 mM Tris (Sigma), 1 mM EDTA; pH 8) with the addition of 100 mM phenylmethylsulfonyl fluoride (Sigma) and 2% SDS (Sigma). The sample was then transferred to MP tube and homogenized using FastPrep-24 MP homogenizer. Subsequently the sample incubated for 30 min at 55°C and centrifuged at 14000 x g at room temperature for 15 min. The supernatant containing proteins was transferred to a new Eppendorf tube and sample was immediately used.

Cell extracts or recombinant protein solutions were subjected to acrylamide electrophoresis. Following protein electrophoresis, the gel (12% Mini-Protean Precast Gel BioRad) was placed in a plastic container and covered with PVDF membrane (ThermoScientific) washed beforehand in methanol and Transfer Buffer (0,2 M glycine (Sigma); 25 mM Tris (Sigma); 20% methanol (Sigma)). The transfer was performed overnight at 4°C at 50 mA in an electrophoresis container filled with transfer buffer. Next the membrane was blocked for 1.5 h at room temperature in PBS (137 mM NaCl (Sigma); 2.7 mM KCl (Sigma); 10 mM Na_2_HPO_4_ • 2 H2O (Sigma); 2M KH_2_PO_4_ (Sigma) pH 7.4) containing 10% milk (Gostynin, fat- free powdered milk). Subsequently, the membrane was incubated in PBS containing 5% milk, 0.05% Tween 20 (Sigma) and primary antibodies (rabbit anti- RnhA, obtained at Department of Immunoparasitology, University of Lodz or rabbit anti- LigA, described previously [44]) at 4°C overnight. Afterwards, the membrane was washed three times in PBS with 0.05% Tween 20 (Sigma). The membrane was then placed in PBS containing 5% milk and a secondary antibody (anti-rabbit goat IgG conjugated with peroxidase (Sigma)). The membrane was incubated for 1 h at room temperature and washed three times with PBS containing 0.05% Tween 20. The membrane was then placed in a dry plastic container and covered with mixture of ECL reagent. Following a 2 minutes incubation the membrane was covered in Saran wrap and exposed to an X- ray film (ThermoScientific) for 2 minutes. The film was developed using Kodak Medical X-ray Processor.

### Statistical analysis

In order to determine growth rates of the analysed strains, we fitted logistic or quadratic curves to the collected measurements of optical density of the liquid cultures. We fitted logistic curves of the form OD = A/[1 + B*exp(-KT)], where OD is optical density at the time T, A is an asymptotic value, B is a constant of integration, and K is the growth rate constant. Parameter K from the fitted curves was used as an indicator of growth rate for each strain. A T-test was used to test for the differences in growth rates between the strains. All statistical analyses were performed with Statistica 10.0 (StatSoft, Tulsa, OK, USA).

## Results and Discussion

### The genome of *M*. *smegmatis* encodes two predicted RNases H type I

**Fig 2:**
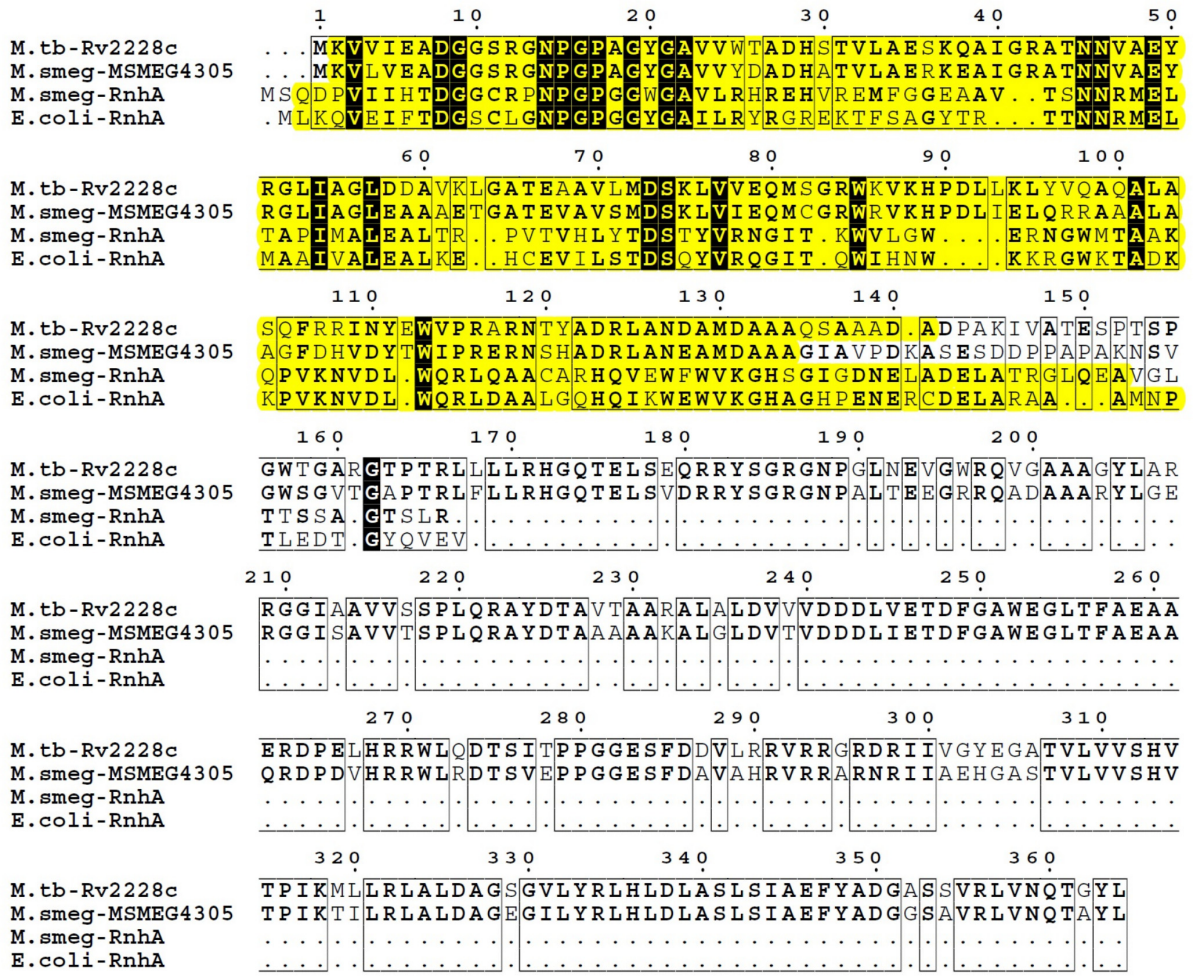
Comparison of protein sequences of RNases H type I. Sequence alignment between RNases H type I of *E*. *coli* K12_MG1655 (RnhA), *M*. *smegmatis* mc^2^ 155 (MSMEG4305 and RnhA) and *M*. *tuberculosis* H37Rv (Rv2228c) was performed using MultiAlin and visualized with ESPript 3.0. Highly similar or identical residues between protein sequences are written in bold. Identical residues across all analyzed sequences are shown in white on a black background. Similarities between protein sequences are marked by framing. The span of RNase H domains in each protein sequence, as defined by SMART, is highlighted in yellow.

Through performing an *in silico* analysis we identified two genes of *M*. *smegmatis* mc^2^ 155 which encode proteins containing RNase HI domain- *rnhA* and MSMEG4305. BLAST analysis of RNase HI domains of RnhA and MSMEG4305 of *M*. *smegmatis* mc^2^ 155 revealed that with 50% of query cover of RnhA, they share 36% of protein sequence identity. Next, MSMEG4305 was shown to be homologous to Rv2228c of *M*. *tuberculosis* H37Rv. Both proteins share 72% sequence identity with 100% query cover of MSMEG4305. RNase HI domains of MSMEG4305 and Rv2228c share 72% identity with 97% of MSMEG4305 query cover, while acid phosphatase domains share 75% sequence identity with 100% query cover of MSMEG4305. The alignment between protein sequences between RNases H type I of *E*. *coli* K12_MG1655, *M*. *smegmatis* mc^2^ 155 and *M*. *tuberculosis* H37Rv is presented in Fig 2.

A possible reason for the coexistence of two RNase H type I encoding genes within the genome of *M*. *smegmatis* is the neofunctionalization of MSMEG4305. Apart from encoding RNase HI domain it also encodes CobC domain involved in vitamin B12 biosynthesis. Such neofunctionalization is thought to increase retention of RNase H type I genes [21]. The coexistence of MSMEG4305 and *rnhA* might be expected to lead to future inactivation of RNase HI domain in one of them and generation of a pseudogene [45]. In fact, the genome of *M*. *tuberculosis*, which is thought to have undergone genome reduction when compared with free-living mycobacteria, contains only a homolog of MSMEG4305.

### Essentiality of RNases H type I in *M*. *smegmatis*

**Fig 3:**
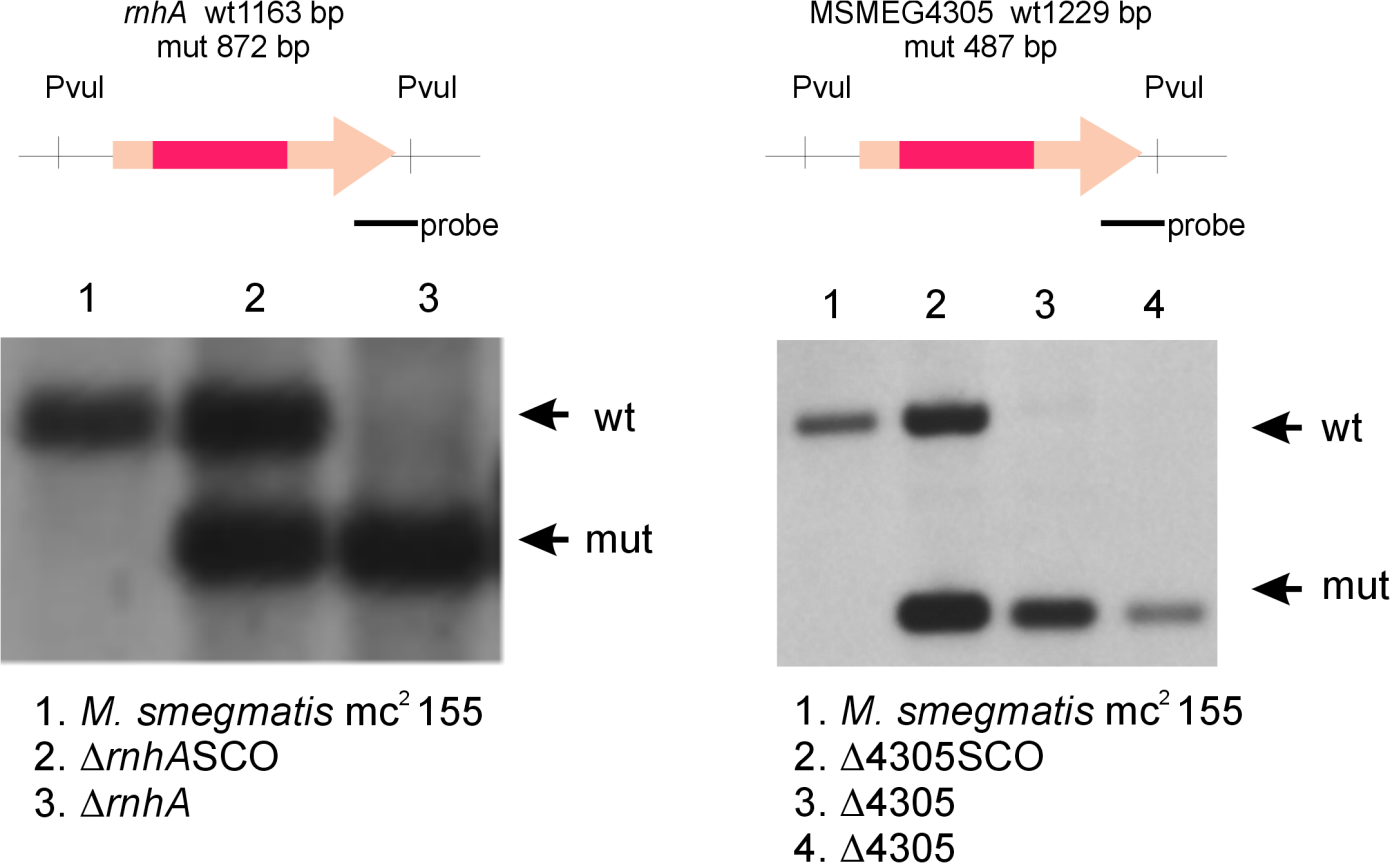
Southern blots confirming deletions in single RNase H type I mutants of *M*. *smegmatis*. We used gene replacement through homologous recombination to obtain mutants deficient in either *rnhA* or MSMEG4305. Briefly, recombinant plasmids containing genomic regions of either *rnhA* or MSMEG4305 with large deletions within each gene were introduced into *M*. *smegmatis* mc^2^ 155 cells. Following multistep selection we were able to identify clones where the native version of each gene has been replaced with manipulated sequence. Intermediate steps of gene replacement procedure are denoted SCO. For more information regarding plasmid construction and gene replacement procedure please refer to the text. Schematic representation of analyzed genomic regions, including enzymes used for digestion, size of restriction fragments following digestion and the site of hybridization of hybridization probe, is presented in the upper part of the figure. Photographic films presenting results of Southern blot analysis are presented in lower part of the figure. Bands corresponding to wild type genotype (wt) and mutant genotype (mut) are marked on the right side of each photograph.

We used the technique of gene replacement through homologous recombination to construct a series of genetic mutants with large deletions introduced within the sequence of predicted RNase H type I encoding genes. We were able to identify single mutants of both *rnhA* and MSMEG4305 (Fig 3). The ability to obtain the mutants signifies that none of these genes itself is essential for survival of *M*. *smegmatis*.

**Fig 4:**
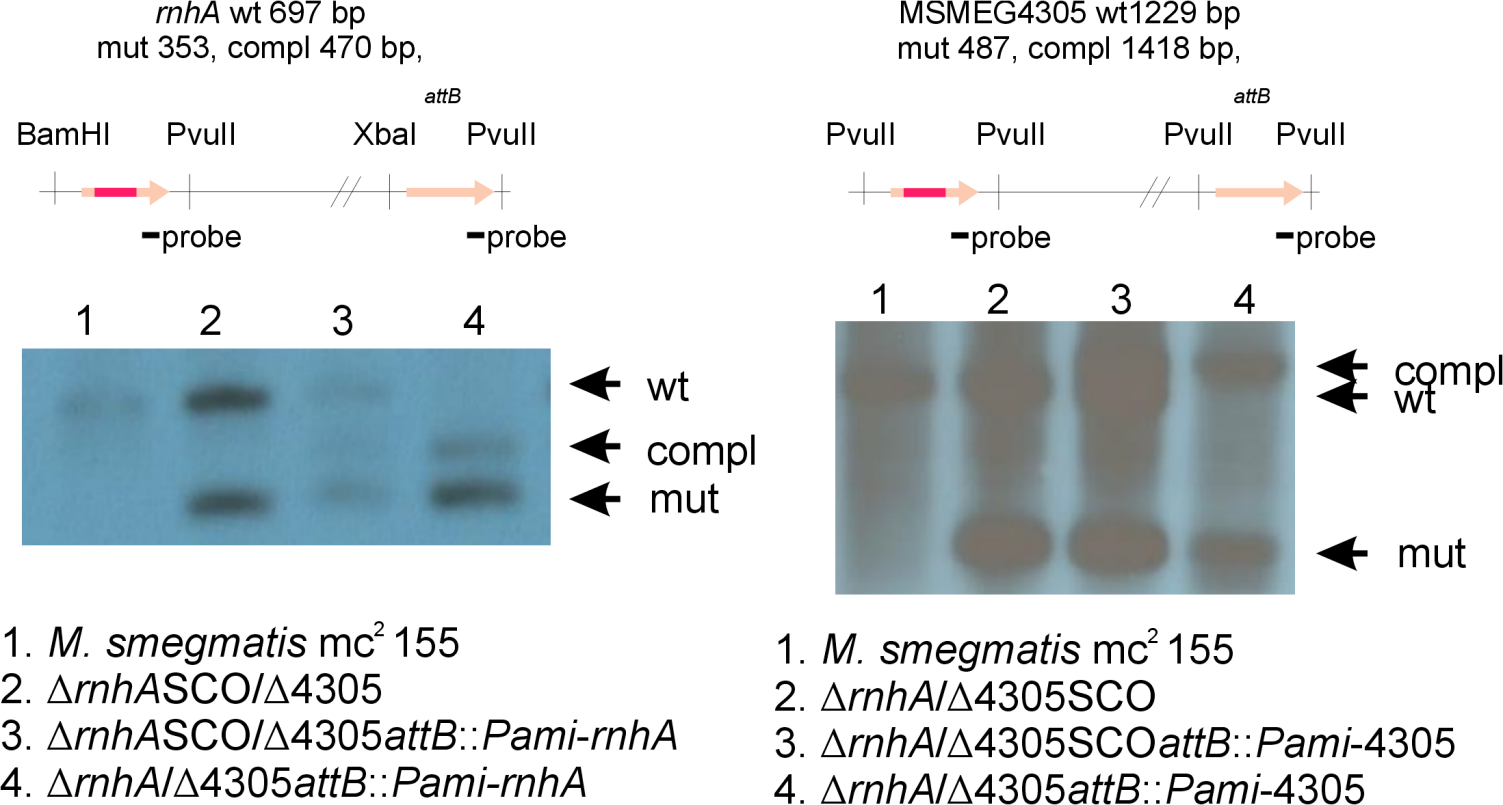
Southern blots confirming deletions in double, complemented RNase H type I mutants of *M*. *smegmatis*. We used gene replacement through homologous recombination to obtain mutants deficient in both *rnhA* and MSMEG4305. We were unable to identify a mutant ∆*rnhA*/∆4305. Therefore, we introduced a functional copy of either MSMEG4305 or *rnhA* at the *attB* site of ∆*rnhA*/SCOMSMEG4305 or SCO*rnhA*/∆MSMEG4305, respectively. Following selection, we generated two strains ∆*rnhA*/∆4305*attB*::*Pami-rnhA* and ∆*rnhA*/∆4305*attB*::*Pami*-4305 deficient for both native versions of RNase HI encoding genes, however complemented with either *rnhA* or MSMEG4305 at the *attB* site. For more information regarding plasmid construction, gene replacement procedure and complementation please refer to the text. Schematic representation of analyzed genomic regions, including enzymes used for digestion, size of restriction fragments following digestion and the site of hybridization of hybridization probe, is presented in the upper part of the figure. Photographic films presenting results of Southern blot analysis are presented in lower part of the figure. Bands corresponding to wild type genotype (wt), mutant genotype (mut) and complementation genotype (compl) are marked on the right side of each photograph.

We were unable to identify a mutant deficient in ∆*rnhA*/∆4305. Therefore, we introduced a functional copy of either MSMEG4305 or *rnhA* at the *attB* site of ∆*rnhA*/SCOMSMEG4305 or SCO*rnhA*/∆MSMEG4305, respectively. Following selection, we generated two strains ∆*rnhA*/∆4305*attB*::*Pami-rnhA* and ∆*rnhA*/∆4305*attB*::*Pami*-4305 deficient for both native versions of RNase HI encoding genes, but complemented with either *rnhA* or MSMEG4305 at the *attB* site (Fig 4). We used these strains to perform ExEV. This experiment greatly increases the number of analyzed cells. It is therefore used as a final confirmation of the essentiality of the gene [46–48]. It also limits drawbacks of gene replacement technique. In our case it was the enrichment of the medium by the substances used as selection markers. Enrichment of medium might be a source of confusion between contradictory reports regarding RNase HI essentiality in *E*. *coli* [29–31]. The authors that have obtained RNase HI deficient mutants stated that the mutants were rich broth sensitive [30,31]. This is thought to be related to high speed of metabolism. High metabolism requires efficient transcription, which leads to generation of many R-loops. R-loops, in turn, cannot be efficiently resolved in the absence of RNase HI and lead to replication fork collapse. In minimal medium, however, the accumulation of R-loops is limited by a slowed down metabolism. Hence they can be either tolerated in the genome or efficiently removed by proteins other than RNase HI [49]. Even after performing ExEV we did not identify any clones devoid of both genes encoding RNases H type I.

The difference between the essentiality of RNase H type I in *M*. *smegmatis* and *E*. *coli* may be explained by genetic background of these species. In *E*. *coli*, *rnhA* encoding RNase H type I has been shown to be synthetically lethal with a number of genes, namely *polA* [50], *recB* [51], *recC* [51] and *recG* [52]. These genes can be found in mycobacterial genome. However, the mutation in *rnhA* has also been shown to be synthetically lethal with mutation in *rep* [53], a homolog of which is absent in the genome of *M*. *smegmatis*. Rep is a helicase involved in restarting DNA replication forks [54], facilitates reannealing of the parental strands during replication [55] and has been shown essential for efficient replication across highly transcribed regions [56,57]. The precise role of Rep remains unknown. Sandler suggested that Rep might be involved in R-loop metabolism and this function, due to synthetic lethality, may be connected with the function of RNase HI [53]. R-loops are linked with transcription and other helicases of *E*. *coli*- RecG [52] and Cas3 [58] are involved in R-loop removal. R-loops, if unresolved, may block DNA replication in several ways. First, unrepaired lesions in displaced DNA strand may become a source of double strand ends and consequent replication fork collapse. Second, RNA hybridized to the DNA may be an obstacle for replication fork progression. Paused replication forks can be cleaved by endonucleases known to cleave recombination intermediates again creating double strand ends [59]. Finally, transcription complex linked to an R-loop might become attached to the hybrid and collide with progressing replication fork. All of these scenarios would result in replication fork collapse and could potentially lead to subsequent cell death [60]. Although the idea that RNase H substrates other than R-loops might be responsible for the lethal phenotype of ∆*rnhA*/∆MSMEG4305 mutant cannot be entirely excluded, it seems unlikely. In other bacteria Okazaki primers have been shown to be removed by other proteins, which are also present within *M*. *smegmatis*, PolI [61–63] and RNase HII [25].

The essentiality of RNase HI domain in *M*. *smegmatis* suggests that similar phenomenon might be present in *M*. *tuberculosis*. Data obtained by high density transposon mutagenesis in *M*. *tuberculosis* seem to confirm this hypothesis [64]. In studies assessing the essentiality of mycobacterial genes by high-density transposon mutagenesis the authors observed a small level of transposon insertions within the gene encoding a homolog of MSMEG4305- Rv2228c. The number of insertions was significantly smaller from what would be expected. Perhaps this observation could be related to insertions within CobC domain encoded by the gene and lethal phenotype in the case of insertions within the region encoding RNase HI domain.

### RNase HI mutants- RnhA level, growth rates and constitutive stable DNA replication

**Fig 5:**
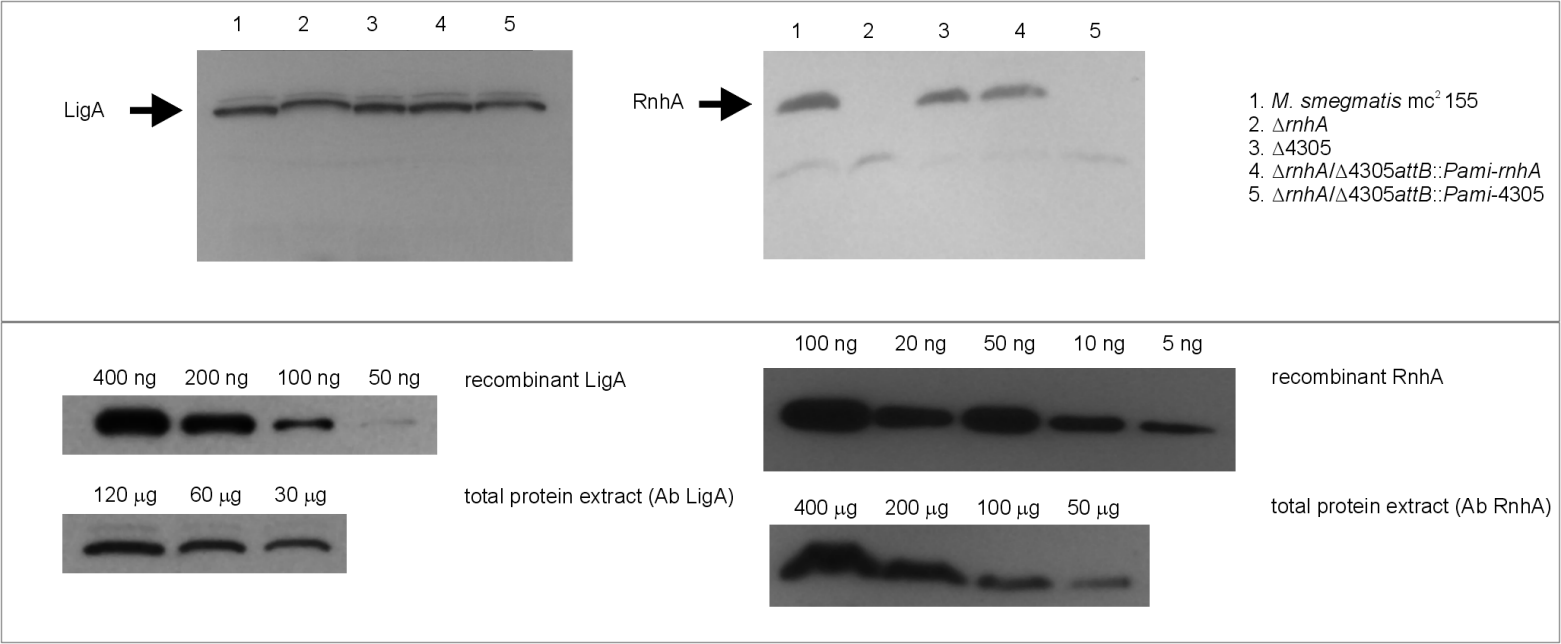
Western blots presenting quantitative analysis of the RnhA protein in protein extracts of *M*. *smegmatis*. The upper part of the figure presents detection of either LigA or RnhA in protein extracts isolated from mutants used in this study. The lower part of the figure presents quantitative analysis of the amount of RnhA in *M*. *smegmatis* mc^2^ 155. Briefly, we detected known concentrations of either recombinant LigA or recombinant RnhA. Further, we compared the intensities of the obtained bands with those obtained from various concentrations of total protein extracts of *M*. *smegmatis* mc^2^ 155. We were able to calculate, that RnhA is approximately 133 times less abundant that LigA.

We wanted to investigate how deletion of MSMEG4305 influences the level of RnhA. Therefore, we obtained recombinant His-tagged RnhA protein expressed in *E*. *coli*. We used this protein to immunize a rabbit and to obtain polyclonal anti-RnhA antibodies. These antibodies were used to perform detection of RnhA in protein extracts isolated from *M*. *smegmatis* strains used in this study. Further, we quantitated the amount of RnhA in *M*. *smegmatis* mc^2^ 155 and compared it with the amount of LigA, NAD^+^ dependent ligase [44]. We observed that the level of RnhA in wild type strain was low (Fig 5). There were 200 ng of LigA detected in approximately 30 μg of total protein extract, while 10 ng of RnhA was identified in approximately 200 μg of total protein extract. Therefore we estimate that LigA is approximately 133 times more abundant than RnhA.

**Fig 6:**
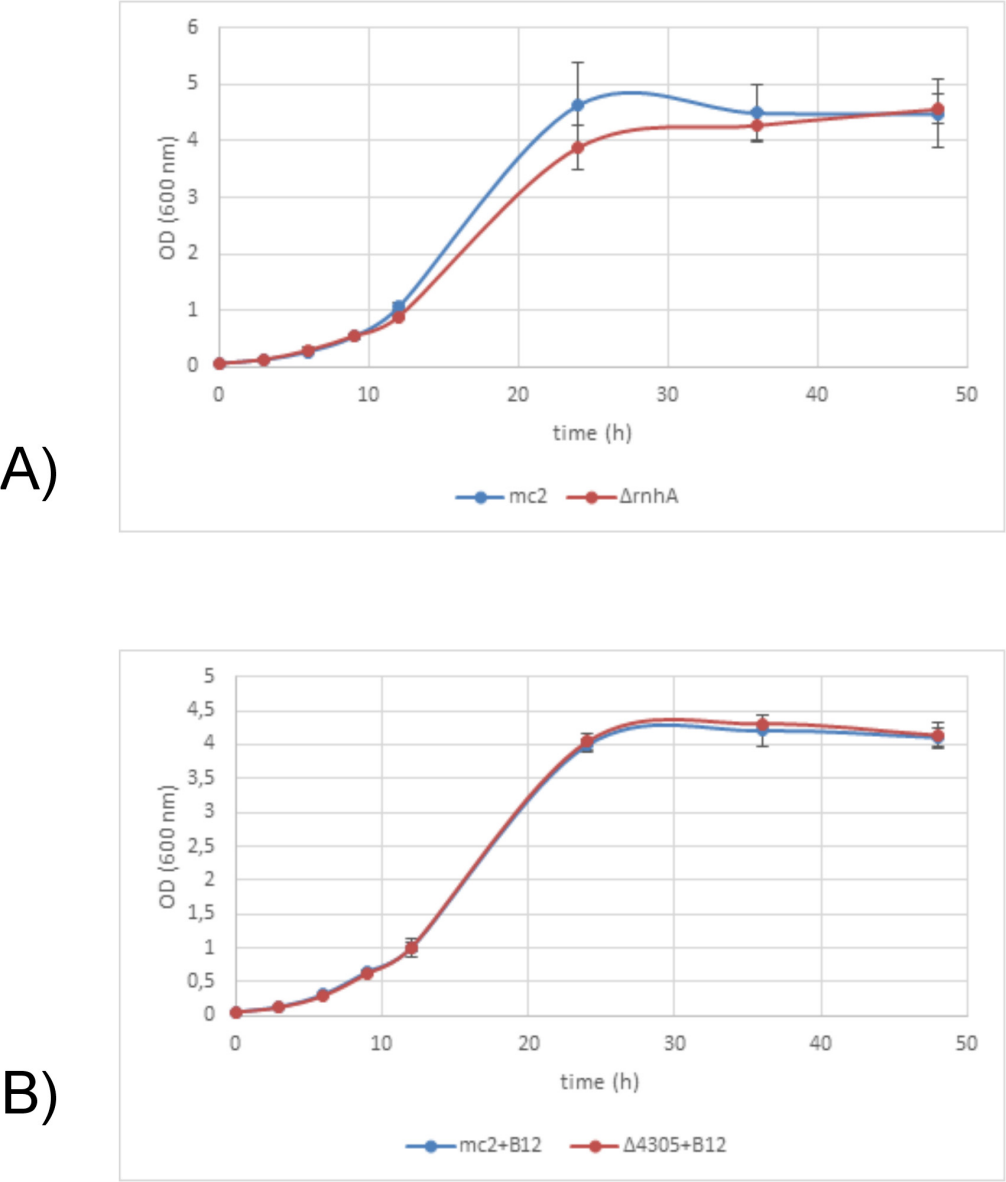
Growth rates of RNase H type I deficient mutants. We analyzed the growth rate of RNase H type I deficient mutants ∆*rnhA* and ∆4305 and compared them with wild type *M*. *smegmatis* mc^2^ 155. The analysis involved measuring optical density of the liquid cultures at determined intervals of time. The cultures were performed on 7H9 medium with the addition of OADC and Tween80. For the comparison between ∆4305 and *M*. *smegmatis* mc^2^ 155 media were additionally supplemented with vitamin B12. Each experiment was performed at least in triplicate. We observed no differences in growth rates based on optical densities of the cultures. (Box- SE, whiskers- 0.95 CI).

**Fig 7:**
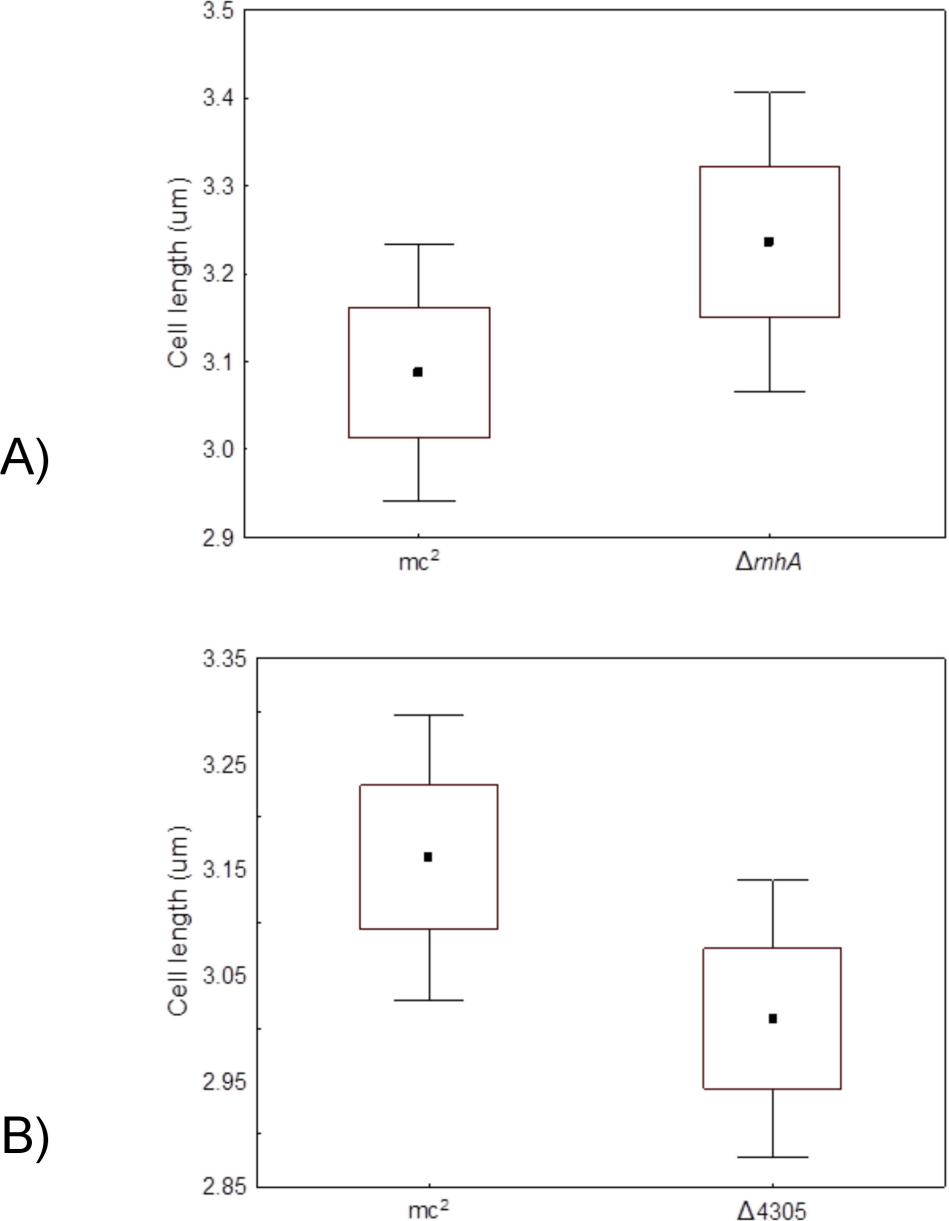
Morphology of the cells of RNase H type I deficient mutants. We harvested cells grown in liquid cultures for 24 hours and analyzed them on Nikon Eclipse TE2000 microscope. The cultures were performed on 7H9 medium with the addition of OADC and Tween80. For the comparison between ∆4305 and *M*. *smegmatis* mc^2^ 155 media were additionally supplemented with vitamin B12. We observed no differences in cell lengths between mutant strains and the wild type. (Box- SE, whiskers- 0.95 CI).

Since the level of RnhA was low, we wanted to see whether deletion of one RNase HI genes influenced growth rate of the mutants. For this purpose, we assessed growth rates by measuring optical density of the liquid cultures at determined intervals of time and fitted appropriate growth curves [65]. We did not observe any differences in growth rates or asymptotical values between the ∆*rnhA* and *M*. *smegmatis* mc^2^ 155 on 7H9 medium supplemented with OADC (t = 1.90, df = 5, p = 0.12; t = 0.35, df = 5, p = 0.74, respectively, data presented in S1 Table) or ∆4305 and *M*. *smegmatis* mc^2^ 155 on 7H9 medium supplemented with OADC and vitamin B12 (t = 0.84, df = 4, p = 0.84; t = 0.49, df = 4, p = 0.65, respectively, data presented in S2 Table) (Fig 6A and 6B). The morphology of the mutant cells grown in before mentioned conditions, in terms of cell length was not altered (t = 1.83, df = 198, p = 0.07 for ∆*rnhA* and *M*. *smegmatis* mc^2^ 155, data presented in S3 Table; t = 1.22, df = 198, p = 0.22 for ∆4305 and *M*. *smegmatis* mc^2^ 155, data presented in S4 Table) (Fig 7A and 7B).

In summary, these results suggest that mycobacterial cells may produce more RNase H type I than would be sufficient for optimal growth, as deletion of one of the genes encoding this enzyme did not seem to affect the viability of the mutant. A similar phenomenon was observed for other replication related proteins of mycobacteria- namely LigA [44] or DnaG [48].

**Fig 8:**
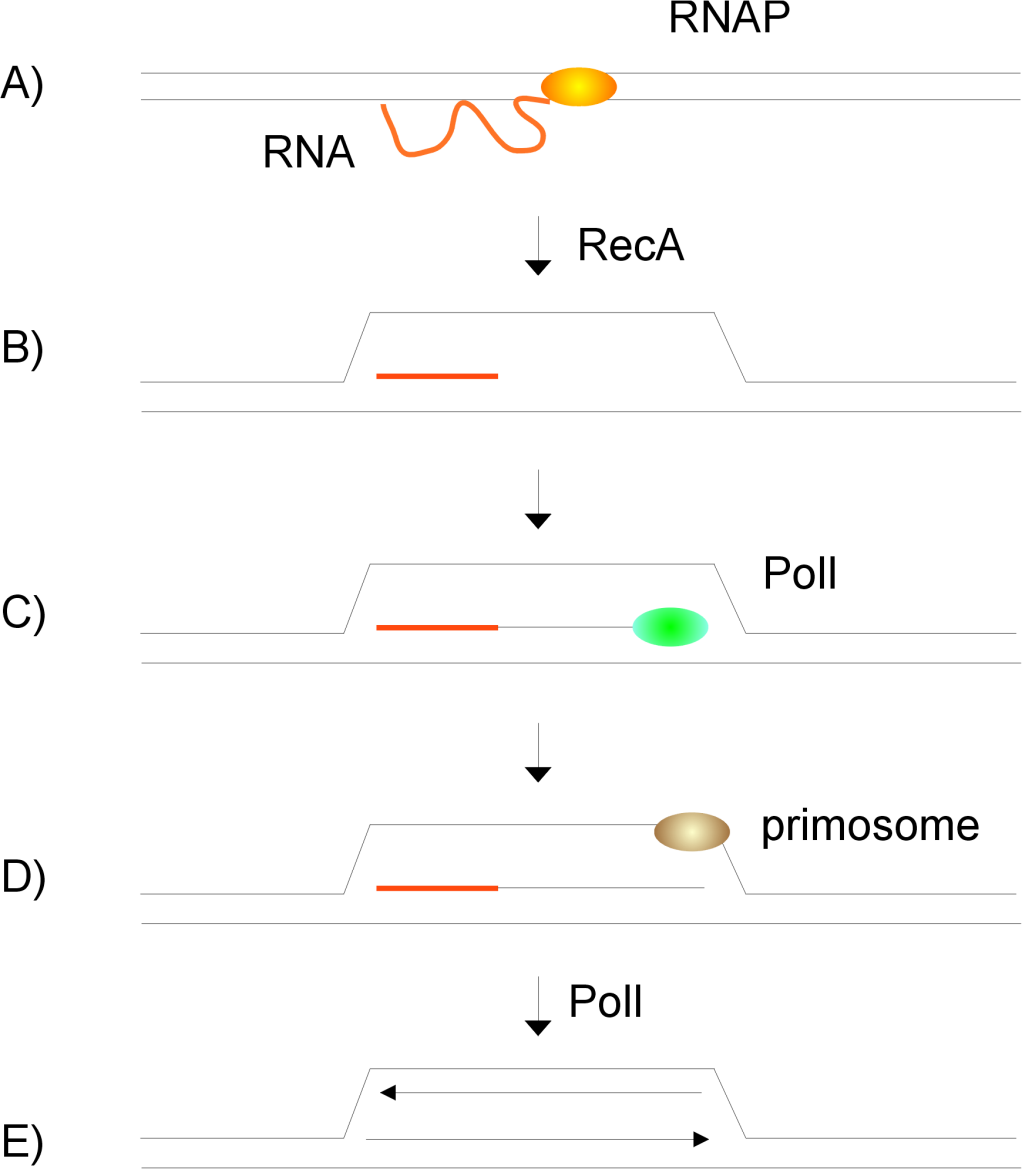
Constitutive stable DNA replication of *E*. *coli* ∆*rnhA* mutants. An alternative mode of initiation of DNA replication was discovered in *E*. *coli* mutants lacking RNase H type I encoded by *rnhA*. (A,B) The initial strand opening involves RecA dependent hybridization of the RNA transcript to dsDNA, (C) The resulting R-loop is not resolved by RNase HI and therefore RNA persists on DNA strand and serves as a primer for elongation by PolI, (D,E) When the loop opens sufficiently, primosome is loaded on the leading strand. As replication continues, PolI removes persisting RNA transcript and primosome is loaded on the lagging strand.

In *E*. *coli* R-loops have been shown to be responsible for alternative pathway of initiation of DNA replication. The phenomenon was termed constitutive stable DNA replication (cSDR). cSDR was identified in thermosensitive *E*. *coli* mutants with inactivated gene encoding RNase HI [66]. Unlike the classical pathway, cSDR initiates from regions termed *oriK* instead of *oriC* [67]. The initial strand opening involves RecA dependent hybridization of the RNA transcript to dsDNA [68] (Fig 8A and 8B). The resulting R-loop is not resolved by RNase HI and therefore RNA persists on DNA strand and serves as a primer for elongation by PolI [50] (Fig 8C). When the loop opens sufficiently, primosome is loaded on the leading strand. As replication continues, PolI removes persisting RNA transcript and primosome is loaded on the lagging strand [50] (Fig 8D and 8E). cSDR in *E*. *coli* is independent of DnaA and *oriC*. Hence, even though *dnaA* and *oriC* are essential for viability of wild type *E*. *coli*, it is possible to obtain double mutants bearing mutations inactivating either *dnaA* and *rnhA* or *oriC* and *rnhA* [69].

**Fig 9:**
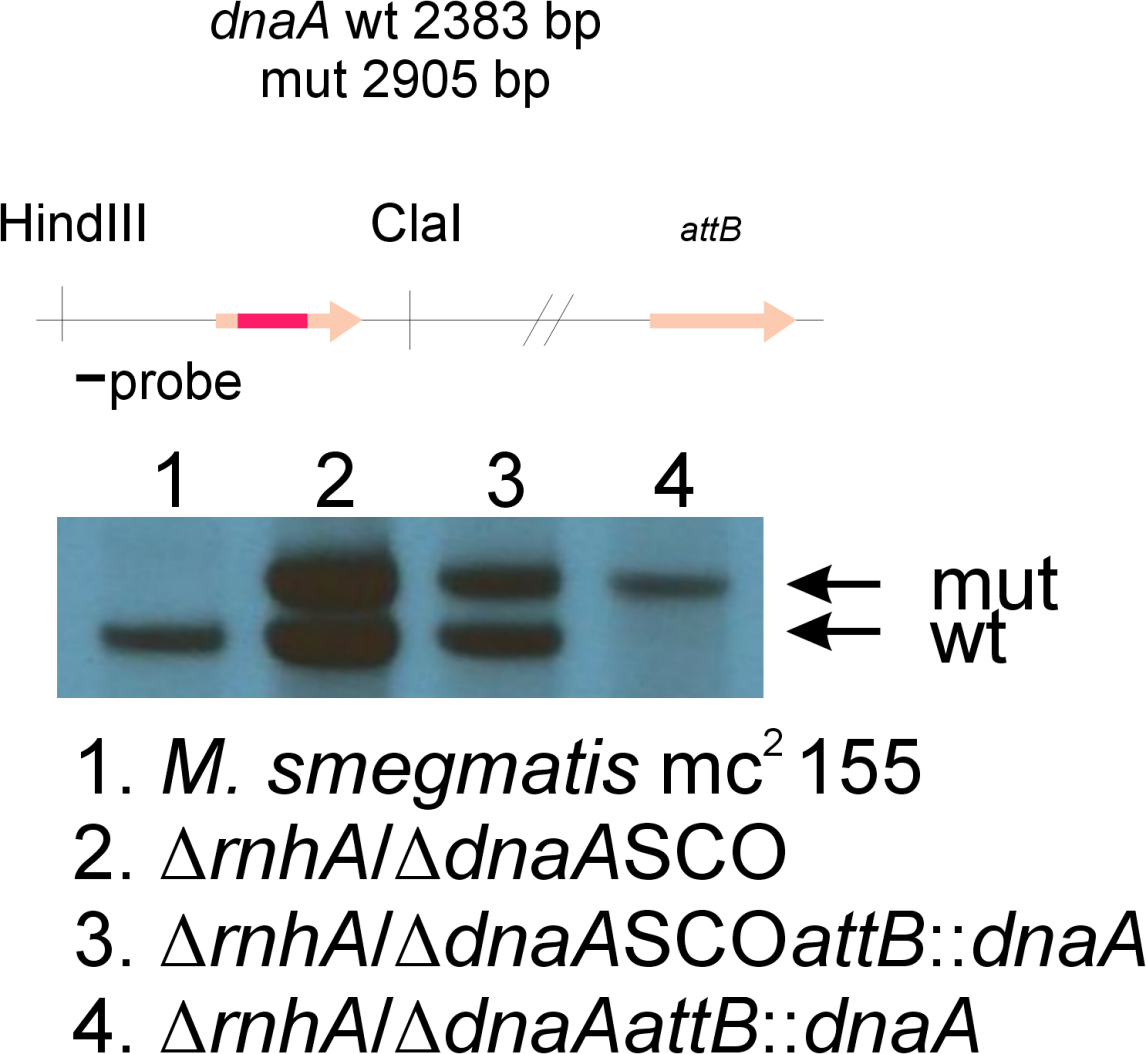
Southern blot confirming deletion of native *dnaA* gene in ∆*rnhA*/∆*dnaAattB*::*dnaA* mutant strain of *M*. *smegmatis*. We used gene replacement through homologous recombination to obtain mutants deficient in *rnhA*. Further, we used the same procedure to generate ∆*rnhA*/*dnaA*SCO strain where SCO signifies an intermediate step of gene replacement procedure. Next, through complementation procedure, we introduced an additional version of *dnaA* gene at the *attB* site of mycobacterial genome. Finally, we removed the native version of *dnaA*, thereby generating a mutant ∆*rnhA*/*dnaA*SCO*attB*::*dnaA*. For more information regarding plasmid construction and gene replacement procedure please refer to the text. Schematic representation of analyzed genomic region, including enzymes used for digestion, size of restriction fragments following digestion and the site of hybridization of hybridization probe, is presented in the upper part of the figure. Photographic film presenting results of Southern blot analysis is presented in the lower part of the figure. Bands corresponding to wild type genotype (wt) and mutant genotype (mut) are marked on the right side of the photograph.

We wanted to investigate whether unaltered growth rate in RNase HI mutants of *M*. *smegmatis* was a consequence of induced cSDR. In order to investigate the presence of cSDR in *M*. *smegmatis* we used the technique of gene replacement through homologous recombination. We introduced gene replacement plasmid for *dnaA*, with Gm resistance cassette cloned within the sequence of the gene to facilitate screening, into ∆*rnhA* and ∆4305. In order to differentiate cell’s metabolism and hence potential level of R-loop formation, we intended to remove the native version of *dnaA* gene on rich medium at 37°C and on minimal medium at 28°C. We were not able to identify a mutant deficient for *dnaA*. To confirm that the plasmid that we used for gene replacement was capable of creating a deletion within the native sequence of *dnaA*, we introduced the complete version of the *dnaA* gene under a native promoter at the *attB* site of ∆*rnhA* strain. We generated a strain, ∆*rnhA*/*dnaA*SCO*attB*::*dnaA*, which was further subjected to gene replacement protocol. We were therefore able to generate a strain deficient for a native copy of *dnaA*, however possessing a copy of the *dnaA* gene at the *attB* site, ∆*rnhA*/∆*dnaAattB*::*dnaA* (Fig 9). Finally, we used the latter strain for ExEV experiment and confirmed that *dnaA* cannot be removed from ∆*rnhA* strain.

The results of this study confirmed that the *dnaA* gene is essential in *M*. *smegmatis* cells in which the formation of R-loops is limited by the presence of both RNases HI, and suggest that *dnaA* gene may be required in cells with an increased tendency to form these loops. However, it cannot be entirely excluded that cSDR is not present under the conditions of our experiment. Further decrease in RNase HI level could affect the ability to remove *dnaA*. Therefore, the existence of cSDR within mycobacteria needs to be further evaluated.

### RNases HI as a new antimycobacterial target?

The products of the genes that are essential for survival of the pathogen are potential targets for novel antibiotics. The possibility that RNase HI might be essential for survival of *M*. *tuberculosis* is particularly interesting as inhibitors of RNase H are currently intensively studied as potential antiviral drugs in HIV therapy [70]. As reported by the World Health Organization, TB is the major cause of death among people living with HIV. Though the idea that one compound might be limiting for both *M*. *tuberculosis* and HIV is wonderful, it is rather utopian. Suboptimal concentrations of RNase H inhibitors of HIV have been shown to decrease susceptibility to antiretroviral drug used for the treatment of HIV, zidovudine [71], and therefore they may be questioned as a novel component of HIV therapy. Finding an RNase H inhibitor that would inhibit both viral and mycobacterial RNase H, without affecting human RNase H, though theoretically possible, does not seem achievable. As an advantage, we observed that in *M*. *smegmatis* RNase HI level was low. Therefore, perhaps even a small amount of an inhibitor could be sufficient to effectively kill these cells. This is particularly important in the case of mycobacteria due to their intracellular life niche (in the case of *M*. *tuberculosis*) and composition of their unusual and thick cell wall. Therefore the question of whether mycobacterial RNases HI may be used for drug development remains open for future research.

## Supporting Information

S1 Table Optical densities of liquid cultures of *M. smegmatis* mc2 155 and ∆*rnhA*. Bacterial cultures were grown on 7H9 media supplemented with OADC and Tween80. At determined intervals of time optical density of the cultures was measured by spectrophotometer (wave length 600 nm). Results were transformed into growth curves.
(XLSX)

S2 Table Optical densities of liquid cultures of *M. smegmatis* mc2 155 and ∆4305. Bacterial cultures were grown on 7H9 media supplemented with OADC, Tween80 and vitamin B12. At determined intervals of time optical density of the cultures was measured by spectrophotometer (wave length 600 nm). Results were transformed into growth curves.
(XLSX)

S3 Table Cell lenghts of *M. smegmatis* mc2 155 and ∆*rnhA*. Bacteria were grown for 24 hours in 7H9 medium supplemented with OADC and Tween80. Cell lengths were measured on Nikon Eclipse TE2000 microscope. Lengths are presented in (nm).
(XLSX)

S4 Table Cell lenghts of *M. smegmatis* mc2 155 and ∆4305. Bacteria were grown for 24 hours in 7H9 medium supplemented with OADC, Tween80 and vitamin B12. Cell lengths were measured on Nikon Eclipse TE2000 microscope. Lengths are presented in (nm).
(XLSX)
